# Right temporal variant frontotemporal dementia is pathologically heterogeneous: a case-series and a systematic review

**DOI:** 10.1186/s40478-021-01229-z

**Published:** 2021-08-03

**Authors:** Hulya Ulugut, Anke A. Dijkstra, Marta Scarioni, Netherlands Brain Bank, Frederik Barkhof, Philip Scheltens, Annemieke J. M. Rozemuller, Yolande A. L. Pijnenburg

**Affiliations:** 1 Alzheimer Center Amsterdam, Department of Neurology, Amsterdam Neuroscience, Vrije Universiteit Amsterdam, Amsterdam UMC, De Boelelaan 1118, 1081 HZ Amsterdam, The Netherlands; 2 Department of Pathology, Vrije Universiteit Amsterdam, Amsterdam UMC, Amsterdam, The Netherlands; 3 Netherlands Institute for Neuroscience, Amsterdam, The Netherlands; 4 Department of Radiology and Nuclear Medicine, Vrije Universiteit Amsterdam, Amsterdam UMC, Amsterdam, The Netherlands; 5 UCL Institutes of Neurology and Healthcare Engineering, University College London, London, UK

**Keywords:** Dementia, Frontotemporal lobar degeneration, Frontotemporal dementia, Right temporal lobe atrophy, Semantic dementia, Pathology, FTLD-TDP, Tauopathies

## Abstract

Although the right temporal variant frontotemporal dementia (rtvFTD) is characterised by distinct clinical and radiological features, its underlying histopathology remains elusive. Being considered a right-sided variant of semantic variant primary progressive aphasia (svPPA), TDP-43 type C pathology has been linked to the syndrome, but this has not been studied in detail in large cohorts. In this case report and systematic review, we report the autopsy results of five subjects diagnosed with rtvFTD from our cohort and 44 single rtvFTD subjects from the literature. Macroscopic pathological evaluation of the combined results revealed that rtvFTD demonstrated either a frontotemporal or temporal evolution, even if the degeneration started in the right temporal lobe initially. FTLD-TDP type C was the most common underlying pathology in rtvFTD, however, in 64% of rtvFTD, other underlying pathologies than FTLD-TDP type C were present, such as Tau-*MAPT* and FTLD-TDP type A and B. Additionally, accompanying motor neuron or corticospinal tract degeneration was observed in 28% of rtvFTD patients. Our results show that in contrast to the general assumption, rtvFTD might not be a pure FTLD-TDP type C disorder, unlike its left temporal counterpart svPPA. Large sample size pathological studies are warranted to understand the diverse pathologies of the right and left temporal variants of frontotemporal dementia.

**Supplementary Information:**

The online version contains supplementary material available at 10.1186/s40478-021-01229-z.

## Introduction

Frontotemporal dementia (FTD) is a neurodegenerative disorder that predominantly affects the frontal and/or temporal lobes. It is subdivided into three different prototypic subtypes; semantic dementia (SD), progressive non-fluent aphasia (PNFA) and behavioural variant frontotemporal dementia (bvFTD) [1]. In 2011, consensus clinical diagnostic criteria were revised and FTD was classified as behavioural variant [2] whereas SD, PNFA and logopenic variant primary progressive aphasia (PPA) were classified under the umbrella of PPA [3]. On the other hand, a number of studies reported a separate syndromic variant that predominantly affects the right temporal lobe (rtvFTD), usually accompanied by behavioural changes, memory deficit and prosopagnosia [4–9]. While rtvFTD cannot formally be considered a PPA variant due to the absence of aphasia, there have been reports of rtvFTD presenting with non-verbal semantic deficits[10] and neuro-radiological studies have shown mirror image findings, suggesting that they might reflect the same pathophysiological process, albeit on opposite sides [3, 11–13].

Pathological examination plays a key role in understanding the nature of the diseases. Unsurprisingly, the neuropathology underlying clinical FTD is also heterogeneous [14]. The term frontotemporal lobar degeneration (FTLD) is used to encompass pathological conditions that present as clinical FTD. FTLD has been classified into four main groups based on the major proteins accumulation in the brain: tau protein (FTLD-tau); TAR DNA-binding protein 43 (FTLD-TDP); ubiquitin positive, TDP-43 negative and immunopositive for the fused in sarcoma protein (FTLD-FUS); and a remaining group encompassing the few cases characterized by inclusions that label only for markers of the ubiquitin proteasome system (FTLD-UPS) or no inclusions [15]. Based on the morphology and cortical distribution of the accumulation, the two main groups (FTLD-tau and FTLD-TDP) have been subdivided; Pick’s disease (PiD), corticobasal degeneration (CBD), progressive supranuclear palsy (PSP), argyrophilic grain disease (AGD), globular glial tauopathy (GGT) and FTD caused by microtubule association protein tau (MAPT) for FTLD-tau [15–18] and the subtypes A, B, C, D and E for FTLD-TDP [19]. These pathological subgroups and their specific pathologies are linked to a number of clinical syndromes. Whereas clinico-pathological concordance is generally weak, particularly for bvFTD, a strong clinicopathological concordance with the underlying FTLD-TDP type C pathology is present in svPPA [20–22].

Since rtvFTD is sometimes considered a type of svPPA [3, 11, 12], FTLD-TDP type C pathology has been linked to the syndrome [13]. Recently, we have described the different clinical progression patterns of rtvFTD and svPPA [9], leading to the question whether their underlying pathologies may differ. To our knowledge, only one post-mortem study has focused on the pathological characteristics of rtvFTD, highlighting the possible association of rtvFTD with underlying tau-pathology [7]. Therefore, we aimed to determine the range of FTLD molecular pathologies underlying the clinical syndrome of rtvFTD based on a combination of clinico-pathological data from the Amsterdam Dementia Cohort and a review of the literature.

## Methods

### Patient selection

**Fig. 1:**
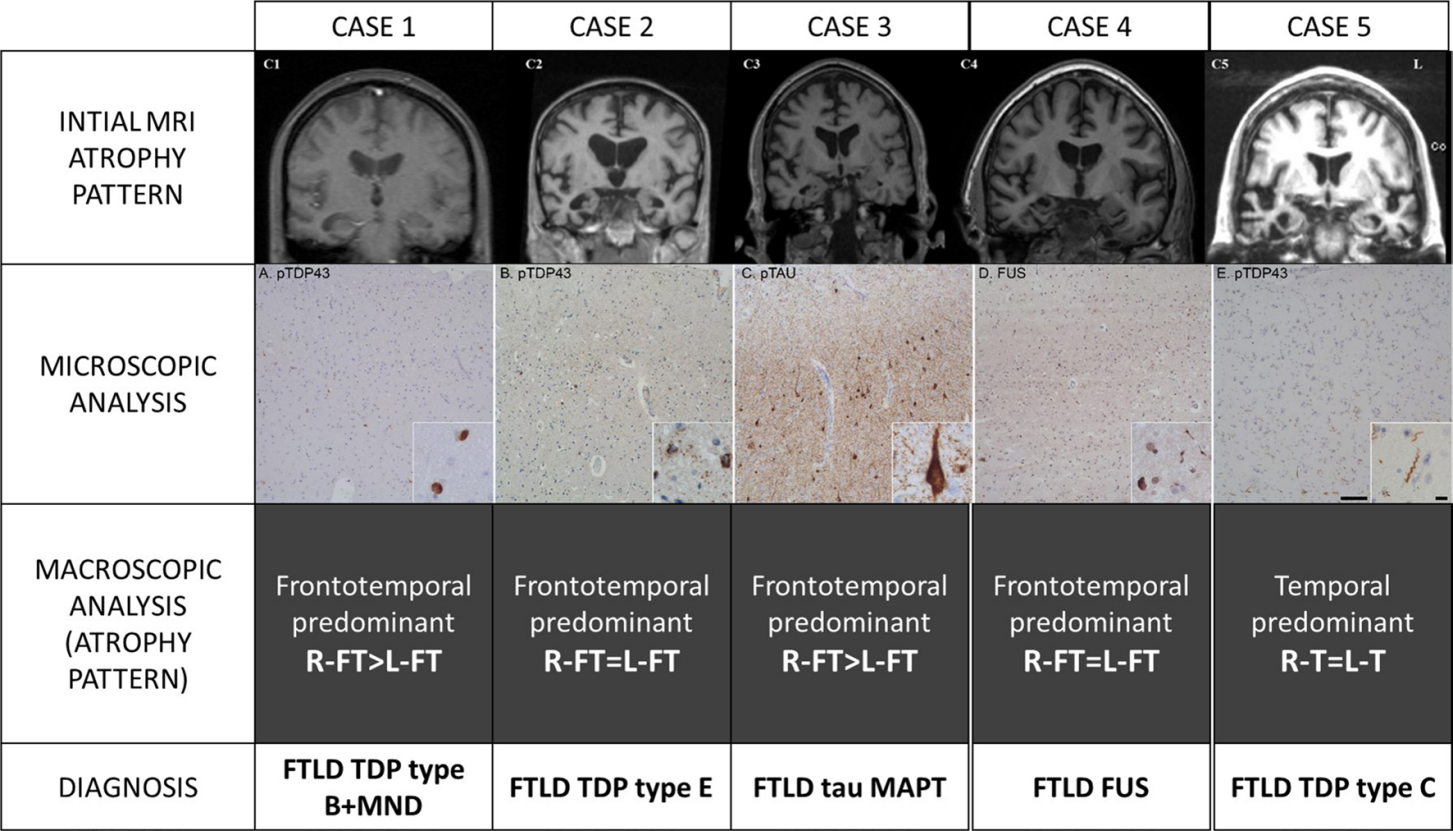
Different pathological diagnoses in donors with rtvFTD. The cases with rtvFTD displayed pathology from different pathological molecular subclasses in FTD. Although all pathological accumulations started from the right temporal lobe, according to the initial MRI atrophy pattern, over time the patients exhibited heterogeneous progression patterns. Case 1 showed FTD-TDP-B pathology with predominant neuronal inclusions throughout the cortical layers in right temporal lobe (**a**). Clinically, motor neuron disease developed over the disease course. Case 2 showed FTD-TDP-E pathology characterised by granulofilamentous neuronal inclusions (insert) and grains in right temporal lobe (**b**). The pathology spread to bilateral fronto-temporal areas. Clinically, this was accompanied by severe behavioural and language problems. Case 3 had tau-pathology with threads and tangles and some plaques (Anterior cingulate cortex: C, adapted from Ulugut Erkoyun et al.,2021, JAD, CC BY-NC 4.0). At the end stage of the disease, right predominant frontotemporal atrophy was observed based on the macroscopic pathological examination. The clinical evolution involved the development of atypical Parkinsonism. Case 4 had large FUS-positive neuronal inclusions and FUS-positive threads (D: right frontal lobe), developed severe global atrophy at a clinical picture of becoming mutistic and bedridden in 4 years after diagnosis. Lastly, case 5 showed long dystrophic neurites characteristic for FTD-TDP-C (E: insular cortex) and developed bilateral temporal atrophy at the end stage of the disease, based on the pathological examination. This patient’s clinical features were relatively benign, presenting with verbal and non-verbal semantic impairment and without the development of any motor disturbances and a disease duration of 12 years. Scalebar is 100 μm, scalebar insert is 10 μm

We identified all subjects diagnosed with FTD and/or PPA from the Amsterdam Dementia Cohort [23] recruited between 1994 and 2019 (n = 669) who had a pathological confirmation of their clinical diagnosis (n = 32) (Ethical approval protocol no: 2016.061). From this group, patients were selected who had a predominant right temporal lobar atrophy on the initial neuroimaging (n = 5) (Fig. 1). In all rtvFTD subjects, the atrophy scores of the right temporal lobe [24–26] were higher (at least 1 grade) than the left temporal lobe and the frontal lobes, as assessed by an experienced neuroradiologist, blinded to the clinical diagnosis (FB). The visual rating scores are displayed in the results section (Table 1). Additionally, in our sample, the frontal atrophy scores were less than grade-1[25] and none of the subjects met the diagnostic criteria of svPPA [3], while all fulfilled at least 2 symptoms out of prosopagnosia, episodic memory impairment, and behavioural change [9], and their clinical profile was in line with the previously reported rtvFTD case series [4, 7, 8] (Fig. 1). Additionally, isolated right temporal lobar hypo-perfusion was reported in Case 1 on perfusion SPECT and isolated right temporal hypometabolism in Case 3 on FDG-PET imaging, in other centres before being referred to us.

**Table 1:** Reclassification of the reported molecular neuropathologies

	Publications	N	Reported molecular neuropathology	Adapted diagnosis
1	[52]	1	FTLD-TDP type C + CTD	FTLD-TDP type C + CTD
2	[69]	1	FTLD-tau-PSP + TDP type A	FTLD-tau-PSP + TDP type A
3	[54]	9	FTLD-tau-PiD (n = 1) FTLD-TDP type C (n = 8)	FTLD-tau-PiD (n = 1) FTLD-TDP type C (n = 8)
4	[61]	1	FTLD-TDP type C + tau-PSP	FTLD-TDP type C + tau-PSP
5	[64]	1	FTLD-TDP type A	FTLD-TDP type A
6	[63]	1	FTLD-TDP type A	FTLD-TDP type A
7	[70]	1	FTLD-tau-PiD	FTLD-tau-PiD
8	[67]	1	TDP-43 pathology in all cortical layers. NCI, with crescentic, round, skein-like and granular types. Short threads accompanied the NCI. Due to the admixture of neuronal cytoplasmic inclusion subtypes seen in FTLD-TDP type A and type B, presence of type A threads, but involvement of all cortical layers (type B), the pattern of TDP-43 inclusions is unclassifiable. Skein-like inclusions in lower motor neurons, producing the neuropathological diagnosis of motor neuron disease. Thal amyloid plaque stage 4, Braak 1. 4R-only atypical tauopathy	FTLD-TDP type A-B + AD + 4R tau
9	[55]	1	FTLD-tau-GGT	FTLD-tau-GGT
10	[38]	7	FTLD-TDP type C (n = 1) FTLD-TDP type C + CTD (n = 6)	FTLD-TDP type C (n = 1) FTLD-TDP type C + CTD (n = 6)
11	[56]	2	FTLD-TDP Mackenzie type 3 + MND (n = 1) TDP Mackenzie type 3 + MND + AD (n = 1)	FTLD TDP type B + MND* FTLD TDP type B + MND* + AD
12	[66]	1	FTLD-FUS	FTLD-FUS
13	[68]	1	FTLD-TDP Mackenzie type 3 + MND	FTLD-TDP type B + MND*
14	[60]	1	FTLD-TDP-43 pathology with NII	FTLD-TDP type A-B
15	[62]	1	FTLD-TDP Cairns type 2 + MND	FTLD-TDP type B + MND*
16	[65]	1	Immunohistochemistry using antibodies to ubiquitin showed NCIs, some of these inclusions were also immunoreactive for phosphorylated TDP-43 antibodies. We identified no DN, but a few NCI, which were positive for both ubiquitin and phosphorylated TDP-43	FTLD-TDP type A-B + MND*
17	[4]	1	Mixed Alzheimer and cortical Lewy body disease (n = 1)	AD + DLB (n = 1)
18	[38]	8	FTLD-tau-PiD (n = 1) FTLD-tau-MAPT (n = 7)	FTLD-tau-PiD (n = 1) FTLD-tau-MAPT (n = 7)
19	[59]	1	TDP-43 pathology with NII	FTLD-TDP type A-B
20	[71]	1	Tau-negative, TDP-43-positive neuronal cytoplasmic inclusions and dystrophic neurites were found. Numerous NFTs and senile plaques with amyloid angiopathy indicated advanced Alzheimer disease	FTLD-TDP type A-B + AD
21	[58]	1	TDP43 pathology with NII + MND	FTLD-TDP type A-B + MND*

TDP: TAR DNA-binding protein 43; TAU: tau protein; MND: motor neuron disease; MAPT: microtubule associated protein; FUS: fused in sarcoma protein; PiD: Pick’s disease; PSP: progressive supranuclear palsy; FTLD-U: frontotemporal lobar degeneration with tau-negative, ubiquitin-immunoreactive pathology; AD: Alzheimer’s disease; DLB: dementia with Lewy bodies; NII: neuronal cytoplasmic and intranuclear inclusions

^*^: Clinically diagnosed with MND

### Clinical and neuropsychological assessment

All 5 rtvFTD subjects had been followed throughout their disease course by an experienced behavioural neurologist. The case notes of all rtvFTD subjects were scrutinized retrospectively. All initial and annual follow-up reports were reviewed by a senior behavioural neurologist (Y.P.) blinded to pathological information. Initial clinical symptoms were collected and family history of any neurodegenerative or psychiatric disease was recorded. The emergence of motor deficits (pyramidal or extrapyramidal) and progression to different clinical syndromes over the disease course was recorded. The following data were extracted of all subjects at the time of initial visit: Clinical Dementia Rating Scale (CDR) [27] and Mini Mental State Examination (MMSE) [28] as global measures, episodic memory [visual association test (VAT) A [29] and the Dutch version of the Rey Auditory Verbal Learning Test (RAVLT)] [30], executive functions [Frontal assessment Battery (FAB) [31], trail making test (TMT) B [32] and digit span backward [33]], language [VAT naming [29]], attention [digit span forward [33] and TMT A [32]] and visuospatial functions [Visual Objective and Space Perception (VOSP)- Dot counting [34]].

### Neuropathological analysis

Subjects were included from the Netherlands Brain Bank and department of pathology, Amsterdam UMC, location Vumc, where tissue was collected according to the local legal and ethical guidelines. All histological slides were re-examined according to the current classification system (A.A.D.) [15, 19]. All pathological examinations were conducted by an expert neuropathologist (A.R.) The pattern of FTLD-TDP pathology was classified into the five following subtypes; A, B, C, D and E [19, 35]. The pattern of FTLD-tau pathology was classified into the six following categories; PiD, PSP, CBD, GGT, AGD and FTD caused by MAPT mutations (tau-MAPT) [15, 18]. Co-existing pathological features such as Alzheimer’s disease (AD) [36], Motor neuron degeneration (MND) [37], corticospinal tract degeneration (CTD) [38] and dementia with Lewy bodies (DLB) [39] were recorded.

Details of the pathological examination are presented in Additional file 1.

### Systematic review

We conducted a systematic review following PRISMA guidelines [40] to identify the papers reporting pathological features of rtvFTD patients with available clinical and neuroimaging data (Additional file 2). The search was completed in December 2019 on two electronic databases; Pubmed and Embase. The following terms were used for the search: ("frontotemporal lobar degeneration" OR "frontotemporal dementia" OR "right temporal " OR “semantic dementia”) AND ("pathology") NOT (“epilepsy” OR “tumor”). No filter was employed in the search. Titles and abstracts of the papers were screened according to the following eligibility criteria:

1. Original research, including case series and individual case reports.
2. Exclusion of review articles and animal studies.
3. Exclusion of reports with insufficient information.

Disagreements on eligibility were resolved through discussion among the authors (Additional file 3).

After detailed screening, 34 studies were eligible for systematic review. Patients with the following diagnoses “right temporal variant FTD”, “right temporal variant semantic dementia”, “right temporal variant svPPA”, “bvFTD presenting with right temporal atrophy”, “right temporal variant bvFTD”, “FTD patient with right temporal atrophy”, “right predominant semantic dementia” were included. Therefore, non-FTD clinical diagnoses such as amyotrophic lateral sclerosis (ALS) or atypical Parkinsonism were excluded. Of note, all case notes and neuroimaging features were also re-assessed. If the left temporal or frontal atrophy was equal or higher than the right temporal atrophy, the subjects were not included. In all included studies, the atrophy pattern had been assessed with either unbiased standardized volumetric morphometry analysis or visual scoring scales. Additionally, in all studies, neuroimaging had been displayed that allowed us to re-assess the radiological features. If detailed radiological information was not eligible, those studies were considered as articles with insufficient information and excluded. Lastly, the clinical features of all cases had been in line with the published rtvFTD literature [4, 8, 9]. Furthermore, all studies were examined in detail to remove cases without TDP-43 staining and when case duplication occurred in, we selected the study from a particular institution/ cohort over a given period of time with the largest sample size. Thirteen studies were excluded based on the criteria mentioned above following author consensus (Additional file 3). This yielded a sample of 21 studies (n = 44) which have defined the molecular pathology in the patients with predominant right temporal atrophy and a consistent clinical syndrome [4, 8, 9] (Table 2). The data from all 44 subjects were combined with our 5 rtvFTD subjects to analyse clinico-pathological associations in rtvFTD.

**Table 2:** Initial clinical features of the rtvFTD subjects

	Case 1	Case 2	Case 3	Case 4	Case 5
Age	58	68	59	59	63
Sex	Male	Male	Male	Male	Female
Handedness	Right	Right	Right	Right	Right
Symptoms					
Prosopagnosia	**√**		**√**		**√**
Memory deficit	**√**	**√**	**√**	**√**	**√**
Disinhibition	**√**	**√**	**√**	**√**	
Apathy-inertia	**√**	**√**	**√**	**√**	**√**
Alexithymia			**√**	**√**	**√**
Bizarre preoccupations	**√**	**√**	**√**	**√**	**√**
Lack of logical reasoning	**√**	**√**	**√**	**√**	**√**
Pathological dwelling on one activity	**√**	**√**	**√**	**√**	**√**
Change of personal taste		**√**	**√**		
Nicotine/alcohol abuse		**√**			
Hyperalgesia			**√**	**√**	
Over sleeping during the day	**√**			**√**	
Word finding difficulties	**√**	**√**		**√**	**√**
Naming difficulties	**√**				**√**
Single word comprehension deficit					**√**
Depression	**√**	**√**	**√**	**√**	**√**
Slowness	**√**	**√**	**√**	**√**	
Motor restless				**√**	
Hyper-orality					
Diagnosis prior to autopsy	FTD + MND	FTD	FTD + atypical parkinsonism	FTD	FTD
Family History	Father had psychiatric symptoms, sister had paranoid disorder	Two brothers and mother had dementia at the age of 70 s with behavioural problems	Mother had psychiatric symptoms and attempt a suicide, uncle (maternal) had dementia at the age of 85	Father and brother had depression, son had ADHD	Mother attempt a suicide
MRI anterior temporal R/L	2/0	3/2	2/0	3/1	4/3
MRI mesial temporal R/L	3/0	4/3	2/1	4/0	3/2
MRI frontal R/L	1/0	1/1	1/0	1/1	1/0
SPECT/PET	Right temporal hypo-perfusion	N.A	Right temporal hypo-perfusion	N.A	N.A
Genetic analysis	N.A	N.A	MAPT (+) Ser352Leu	N.A	*MAPT* (negative) *PRGN* (negative) *C9orf72* (negative)
CDR	0.5	0.5	0.5	0.5	1
MMSE	27/30	25/30	23/30	22/30	25/30
FAB	N.A	N.A	14/18	18/18	14/18
VAT-A	N.A	6/12	4/12	4/12	7/12
RAVLT delayed recall	N.A	0/15	N.A	12/15	N.A
VAT naming	N.A	N.A	10/12	12/12	6/12
Digit span forward	N.A	N.A	12/16	13/16	8/16
Digit span backward	N.A	N.A	8/16	7/16	8/16
TMT A	N.A	N.A	57’’ (A)	69’’ (A)	49’’ (A)
TMT B	N.A	N.A	169’’ (LA)	166’’ (LA)	102’’ (A)
VOSP-Dot Counting	N.A	N.A	10/10	8/10	9/10

rtvFTD: right temporal variant frontotemporal dementia; bvFTD: behavioural variant frontotemporal dementia; svPPA: semantic variant primary progressive aphasia; MND: motor neuron disease; ADHD: attention deficit hyperactivity disorder; FTD; frontotemporal dementia, CDR; Clinical dementia rating, MMSE; mini-mental state examination, VAT; visual association test, RAVLT; Dutch version of the Rey Auditory Verbal Learning Test, FAB; frontal assessment battery, TMT; trial making test, VOSP; Visual objective and space perception, A; Avarage, LA; low average, N.A.; not available

Since the classification of the molecular neuropathology of FTD has been updated over the years, we adapted all reviewed pathology reports based on the current classification system and the subtype nomenclature used was that of the more recent harmonized classification system; FTLD-TDP type A = Mackenzie type 1/Sampathu type 3, type B = Mackenzie type 3/Sampathu type 2, type C = Mackenzie type 2/Sampathu type 1, type D = type 4 with VCP mutations [15, 35]. There was no correction for the FTLD-TDP type E diagnosis [19]. In a subset of cases, however available pathological data were insufficient to identify either TDP type A or B. These cases were denominated as TDP- A-B (Table 2).

## Results

### Demographic and clinical data of our cohort

All Amsterdam cases were right-handed. The rtvFTD group comprised 4 male and 1 female patients. Demographic data, detailed clinical symptoms and cognitive test results are displayed in Table 1. All subjects had behavioural problems, depression and memory deficits. While 3 of them had prosopagnosia, 4 of them had word finding difficulties. Additionally, they became negativistic, non-flexible, sensitive to pain, very fixated on certain thoughts or activities, and they lost their logical reasoning. For instance, due to drinking while driving, Case 2’s driver’s license was withdrawn, which means that he could no longer be a volunteer for the Red Cross. Interestingly, while he did not care for his driving licence, he became obsessed with working in the Red Cross. On the other hand, Case 3 decided to be the golf champion in the Netherlands and spent his entire time and money for this sport, even though he became extremely stingy regarding other daily life activities, including costs for showering. Other cases also displayed bizarre rituals such as walking/cycling for miles in the same route every day or repeating the same eating/drinking routine etc. Change of personal taste (food, colours, music etc.) was another prominent feature. Importantly, their behavioural profile was quite different from bvFTD [2], and they had several non-verbal semantic deficits that might cause those behavioural-psychiatric problems. Furthermore, unlike svPPA, aphasia was not the most prominent feature and neither svPPA diagnostic criteria covered their initial symptom distribution [3], however their clinical phenotypes were in line with the published rtvFTD literature [4, 8, 9]. Although rtvFTD cases had fairly similar initial clinical presentations, over the years, they exhibited a different progression pattern. While the clinical diagnosis of three of the cases remained FTD, one of the cases (Case 1) developed concomitant MND, whereas another patient carrying a heterozygous *Ser352Leu* mutation in the MAPT gene developed atypical parkinsonism (Case 3) (Table 1). The underlying genetics of this case have been published recently [41].

### Pathological features of our cohort

Details of the pathological results of the Amsterdam subjects are displayed in Table 3. The rtvFTD group exhibited a heterogeneous underlying pathology, including FTLD- TDP type B with motor neuron degeneration, FTLD-TDP type E, FTLD- MAPT, FTLD-FUS, and FTLD-TDP type C (Fig. 1). The macroscopic analysis revealed that except Case 5, who had an underlying TDP- C pathology and a predominant bilateral temporal atrophy, all rtvFTD cases had either right predominant or bilateral frontotemporal involvement at the end stage of the disease. (Table 4).

**Table 3:** Pathological features of rtvFTD cases

	Case 1	Case 2	Case 3	Case 4	Case 5
Macroscopic analysis					
Brain weight	1117 gr	1410 gr	1260 gr	1010 gr	975 gr
Atrophy	FT-Right	FT	FT-Right	FT	T
Substantia nigra	Normally pigmented	Normally pigmented	Slightly pale	Pale	Slightly pale
Locus coeruleus	Visible	Visible	Right < Left	Not visible	Visible
Atherosclerosis	No	Moderate	Severe	Mild	No
Microscopic analysis					
Plaque and tangles	Negative	Negative	Thal 3	Negative	Negative
Congo red	Negative	Negative	Negative	Negative	Negative
Alpha synuclein	Negative	Negative	Negative	Braak 3	Negative
Tau	Negative	Negative	Positive	Negative	Negative
Pick Bodies	No	No	No	No	No
TDP-43	Positive	Positive	Negative	Negative	Positive
FUS	Negative	Negative	Negative	Positive	Negative
Accumulation	All layers	Predominantly layer 2	3R + 4R	FUS	Several long threads
Frontal	+ +	+ +	+ + +	+ + +	+
Temporal	+ + +	+ + +	+ + + *	+ + +	+ + +
Motor cortex	+ + +	–	–	n/a	–
Corticospinal tract	+ + +	–	–	–	–
Parietal	–	–	+ +	n/a	+
Occipital	–	–	+	–	–
Hippocampus	+ + +	+ + +	+ + +	+ + +	+ + +
Amygdala	+ + +	+ + +	+ + +	+ + +	+ + +
Caudate, putamen	–	+ +	+ + +	+ + +	+ +
Thalamus	–	+ +	+ +	+ + +	+ +
Brain stem	–	–	+ + +	+ + +	–
Cerebellum	–	–	–	–	–
Cervical cord	+ + +	–	–	–	–
Diagnosis	FTLD-TDP type B + MND	FTLD-TDP type E	FTLD-MAPT	FTLD-FUS	FTLD-TDP type C

rtvFTD: Right temporal variant frontotemporal dementia, FTLD: Frontotemporal lobar degeneration, MAPT: Microtubule associated protein tau, TDP-43: TAR DNA-binding protein 43, n/a: not available, F: Frontal, T: Temporal, R: Repeat

*Extensive tau positivity indicates a primary tauopathy. Pathological results are suggestive for tau mutation

+++: Severe, ++: Moderate, +: Mild, –: Normal

**Table 4:** Pathological features of diagnostic groups

Case	Diagnosis	Macroscopic analysis (atrophy pattern)	Microscopic analysis
1	rtvFTD	Frontotemporal predominant R-FT > L-FT	FTLD-TDP type B + MND
2	rtvFTD	Frontotemporal predominant R-FT = L-FT	FTLD-TDP type E
3	rtvFTD	Frontotemporal predominant R-FT > L-FT	FTLD-tau-MAPT
4	rtvFTD	Frontotemporal predominant R-FT = L-FT	FTLD-FUS
5	rtvFTD	Temporal predominant R-T = L–T	FTLD-TDP type C

rtvFTD: right temporal variant frontotemporal dementia; svPPA: semantic variant primary progressive aphasia; R: right; L: left; F: frontal; T: temporal; TDP: TAR DNA-binding protein 43; TAU: tau protein; MND: motor neuron disease; MAPT: microtubule associated protein; FUS: fused in sarcoma protein; PiD: Pick’s disease

### Systematic review

**Fig. 2:**
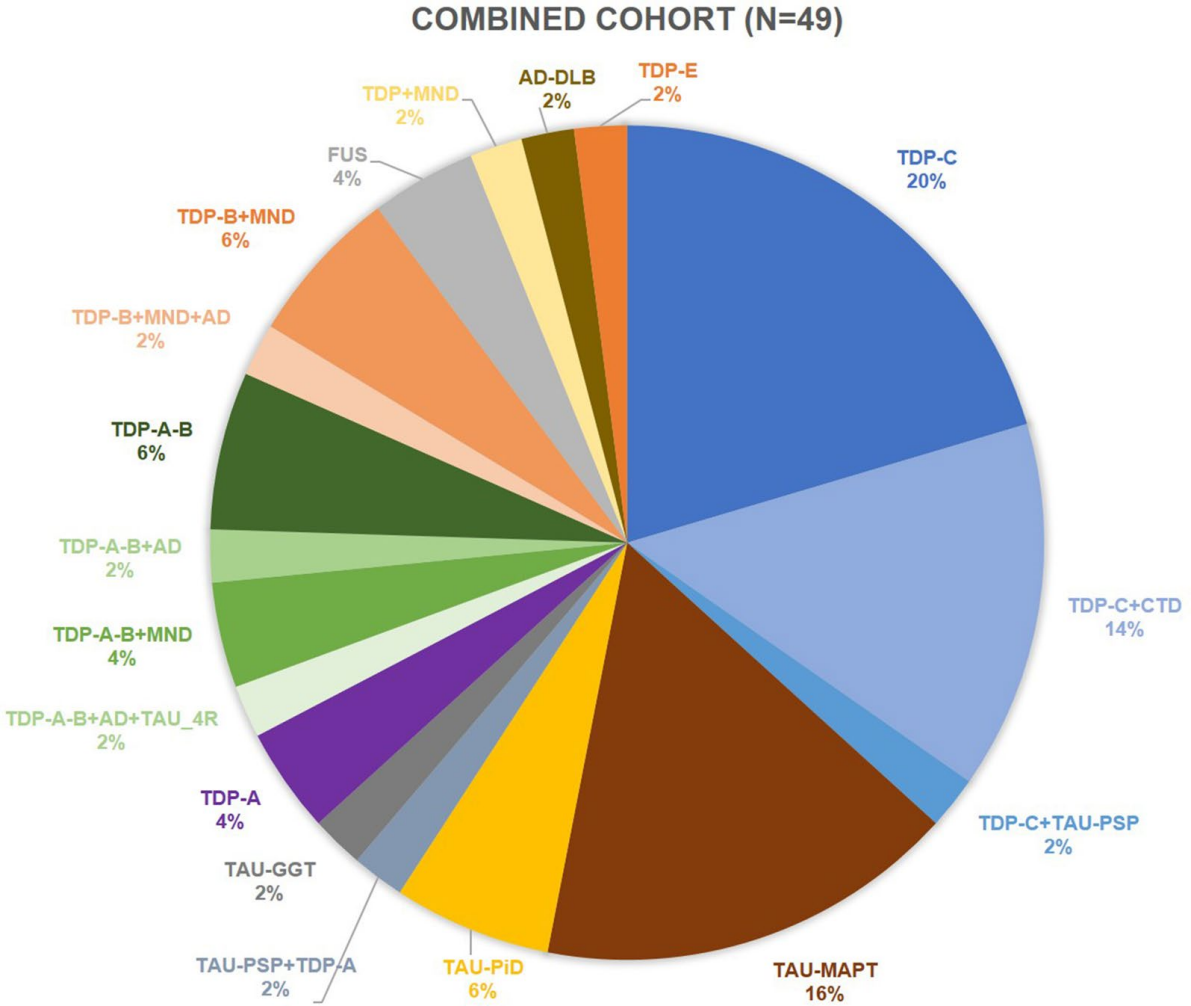
Molecular pathological features of right temporal variant frontotemporal dementia. TDP: TAR DNA-binding protein 43; TAU: tau protein; MND: motor neuron disease; CTD; corticospinal tract degeneration; MAPT: microtubule associated protein; PiD: Pick’s disease; PSP: progressive supranuclear palsy; GGT: globular glial taupathy; FUS: fused in sarcoma protein; DLB: dementia with Lewy bodies; AD: Alzheimer’s disease

The pathological data of 21 studies from 13 centres could be pooled and various molecular neuropathological associations were observed (Table 5). The combination of our results and the results of the systematic review revealed that the underlying pathology of rtvFTD (n = 49) was heterogeneous (Fig. 2). The two most common underlying pathologies in rtvFTD were FTLD-TDP (67.3%) and FTLD-tau (26.5%). The observed FTLD-TDP subtypes were FTLD-TDP type C (36.7%), type B (10.2%), type A (4.1%), type E (2%), whereas 16.3% of cases were labelled as FTLD-TDP type A-B. Despite the relatively high frequency of FTLD-TDP type C pathology, 7 out of 18 FTLD-TDP type C subjects had a CTD co-pathology and one subject diagnosed with FTLD-TDP type C had a tau-PSP co-pathology. In other FTLD-TDP sub-groups, co-pathologies such as MND, tau and AD also occurred (Fig. 2). The observed FTLD-tau subtypes were tau-MAPT (16.3%), tau-PiD (6.1%), tau-GGT (2%) and tau-PSP (2%). The minority of the subjects was diagnosed with FTLD-FUS (4.1%) and only one subject had concomitant AD and DLB pathology.

**Table 5:** Outcomes of the included studies

	Publications	N	Institution	Country	Macroscopy (atrophy pattern)	**Microscopy**
1	[52]	1	UCL	UK	Frontotemporal predominant R-FT = L-FT	TDP type C + CTD
2	[69]	1	MCCN	UK	Temporal predominant R-T = L–T	Tau-PSP + TDP type A
3	[54]	9	UCSF	USA	Frontotemporal predominant R-FT = L-FT (n = 1) TDP-C: N.A	Tau-PiD (n = 1) TDP type C (n = 8)
4	[61]	1	UCSF	USA	N.A	TDP type C + Tau-PSP
5	[63]	1	UCL	UK	Temporal predominant R-T = L–T	TDP type A
6	[64]	1	Helsinki University	Finland	Frontotemporal predominant R-FT = L-FT	TDP type A
7	[70]	1	Cambridge Brain Bank	UK	Temporal predominant R-T = L–T	Tau-MAPT
8	[55]	1	UCL	UK	Frontotemporal predominant R-FT > L-FT	Tau-GGT
9	[67]	1	UCSF	USA	Frontotemporal predominant R-FT > L-FT	TDP type A-B + AD + 4R tau
10	[38]	7	Mayo Clinic	USA	Individual data N.A. Overall, temporal predominant	TDP type C (n = 1) TDP type C + CTD (n = 6)
11	[56]	2	Mayo Clinic	USA	N.A	TDP type B + MND* (n = 1) TDP type B + MND* + AD (n = 1)
12	[66]	1	UCSF	USA	Striatal predominant	FUS
13	[68]	1	Uppsala University	Sweden	Temporal predominant R-T > L–T	TDP type B + MND*
14	[60]	1	UCL	UK	N.A	TDP type A-B
15	[62]	1	Tokyo IP	Japan	Temporal predominant R-T > L–T	TDP type B + MND*
16	[65]	1	Tokyo IP	Japan	Temporal predominant R-T > L–T	TDP type A-B + MND*
17	[4]	1	UCL	UK	N.A	AD + DLB (n = 1)
18	[38]	8	Mayo clinic	USA	N.A	Tau-PiD (n = 1) Tau-MAPT (n = 7)
19	[59]	2	UCL	UK	N.A	TDP type A-B (n = 2)
20	[71]	1	Aichi University	Japan	Frontotemporal predominant R-FT = L-FT	TDP type A-B + AD
21	[58]	1	Northwestern University	USA	N.A	TDP type A-B + MND*

TDP: TAR DNA-binding protein 43; TAU: tau protein; CTD: corticospinal tract degeneration; MND: motor neuron disease; MAPT: microtubule associated protein; FUS: fused in sarcoma protein; PiD: Pick’s disease; PSP: progressive supranuclear palsy; FTLD-U: frontotemporal lobar degeneration with tau-negative, ubiquitin-immunoreactive pathology; AD: Alzheimer’s disease; DLB: dementia with Lewy bodies; UCL: University College London; UCSF: University of California San Francisco; FTD: frontotemporal dementia; MCCN: Manchester Centre for Clinical Neurosciences; IP: institute of psychiatry; VAPSHCS: Veterans Affairs Puget Sound Health Care System

*Clinically diagnosed with FTD + MND

**Fig. 3:**
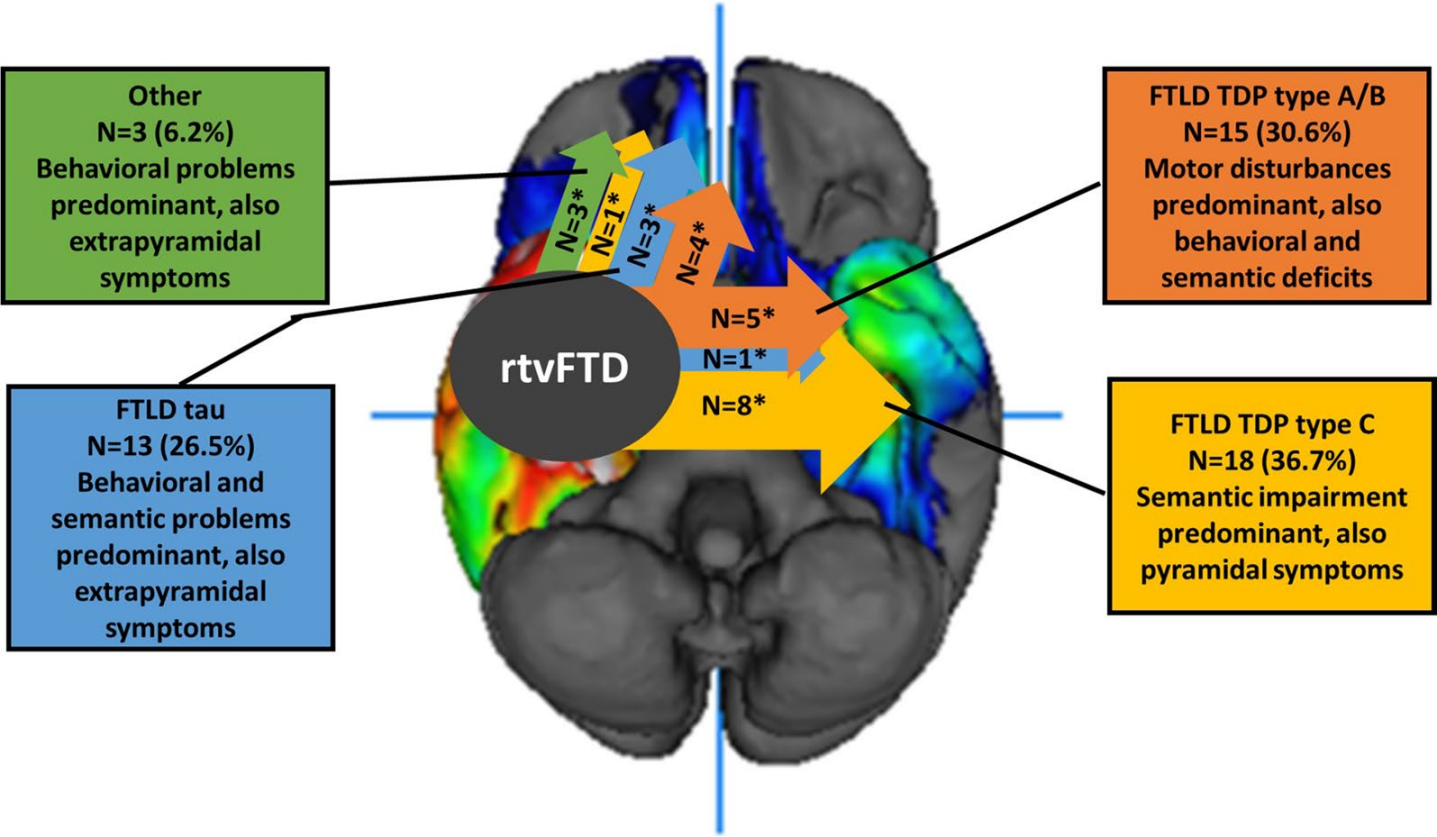
A schematic summary on the FTLD pathologies and symptoms related with rtvFTD. Adapted from Ulugut Erkoyun et al., 2020, Brain, CC BY-NC 4.0. The most common pathological accumulations in rtvFTD were FTLD-TDP type C, FTLD-tau and FTLD TDP type A or B. Since the pathology starts in the right temporal area, initial clinical features were right temporal lobe related symptoms. However, the progression pattern was heterogeneous in rtvFTD. While FTLD-TDP type C mostly spread to the contralateral temporal lobe and the clinical features were related with semantic impairment, FTLD-tau tended to spread to frontal areas, and patients developed more behavioural problems. FTLD-TDP type A/B had a strong relationship with pyramidal impairment. However, in rtvFTD, corticospinal tract impairment was common in FTLD-TDP type C pathology as well and atypical Parkinsonism might be expected in FTLD-tau cases. *: number of cases that have macroscopic atrophy pattern data

Macroscopic findings were reported in 14 out of 21 studies. The combination of our results and the literature (n = 25) revealed that the macroscopic atrophy pattern was again heterogeneous in rtvFTD. Frontotemporal predominant involvement was reported in 11 out of 25 subjects whereas 14 exhibited a temporal predominant atrophy pattern. One FUS case had a striatal predominant atrophy pattern alongside frontotemporal atrophy. Whereas 8 out of 9 TDP type C cases had temporal predominant atrophy in the macroscopic examination, other subtypes such as FTLD-tau or TDP type A-B had either temporal predominant atrophy at the end stage of the disease. Of note, macroscopic atrophy results were available only in 4 tau and 9 TDP type A or B cases (Fig. 3).

## Discussion

In this case series and systematic review, we ascertained the heterogeneous underlying molecular neuropathology of rtvFTD, showing that it cannot be considered a pure FTLD-TDP type C syndrome. In rtvFTD, the most common underlying pathologies were FTLD-TDP type C, tau-MAPT as well as TDP type A and B, whereas its left temporal counterpart; svPPA links to the TDP type C pathology. Moreover, accompanying MND or CTD was prominent in rtvFTD, whereas this has not been reported in larger studies on svPPA [20–22]. Furthermore, the macroscopic descriptions revealed that although neurodegeneration started in the right temporal lobe according to initial neuroimaging, atrophy spread to either the frontal areas or left temporal area which might be the explanation of the heterogeneous clinical progression pattern in rtvFTD.

The systematic review showed that TDP type C pathology was the most common underlying pathology of rtvFTD. Still in the combined dataset, it was observed in only a third of rtvFTD patients and approximately half of them had a co-pathology such as CTD and tau-PSP.

Following the FTLD-TDP type C diagnosis, the second most common pathological diagnosis of rtvFTD was FTLD-*MAPT*. This result might be expected because the association between tau mutations and anterior temporal atrophy is well known [42–45] and genetic studies have shown the relationship between *tau* mutations and rtvFTD [7, 41]. However, the relationship between specific right temporal atrophy and tau mutations is still unknown. According to previous studies, FTLD-*MAPT* exhibits a symmetrical atrophy pattern, despite the fact that clinically, the most common tau mutations produce behavioural symptoms and later semantic impairment [42, 44] which resembles the clinical profile of rtvFTD [9]. Additionally, the association between svPPA and *MAPT* mutations is quite rare [22, 46–48]. Moreover, a recent GENFI paper reported that in the pre-symptomatic carriers of the *MAPT*, *GRN* and *C9orf72* genes, there was significant evidence of atrophy in the right anterior insula and they suggested that there may be some distinct regions in which the disease process starts [49]. This may explain the pathological diversity between two temporal lobe disorders; rtvFTD and svPPA. Future studies combining neurodevelopmental, embryonic, clinical, genetic and pathological findings will be required to further understand the biological basis of selective and lateralized neurodegeneration.

It has previously been suggested that rtvFTD can be divided into two major subtypes; the semantic clinical phenotype associated with temporal atrophy and TDP type C pathology and the behavioural type associated with frontal atrophy and FTLD-*MAPT *[7]*.* Even though our study confirms the observation of two anatomical rtvFTD variants, we argue that the motor component of the syndrome should not be neglected. However, due to low case numbers, we cannot derive associations with specific types of underlying pathology. Future larger dataset studies are warranted to elucidate the underlying pathology specific clinical presentation and progression pattern in rtvFTD.

In contrast to the previous argument, Borghesani et al., (2020) suggested that the left and the right temporal variant of FTD should be considered the same disease based on their similar neuroanatomical progression patterns within the temporal and contralateral temporal regions[50]. However, the limitation of that study is that only subjects with TDP type C pathology were included, thereby potentially excluding other underlying pathologies with a different progression pattern. Additionally, it must be noted that most neuropathological studies taking into account the underlying neuropathology of rtvFTD were based on svPPA cohorts[20, 50], hence reports of underlying pathology of rtvFTD diagnosed with bvFTD are lacking.

One of the important results of our study is the relationship between rtvFTD and co-existing MND or CTD features. Co-existing CTD or MND was observed in 28.6% of rtvFTD subjects in our combined dataset. The general assumption is that ALS links to either bvFTD or nfvPPA while the association with svPPA is rare [51]. In addition, although previous pathological studies have revealed that those accompanying pathologies are mostly related with either FTLD-TDP type B or A- B subtypes [35], our results point out that CTD might accompany FTLD-TDP type C, in particular in rtvFTD. This association was also suggested by Josephs and colleagues (2013) [7]. Of note, some authors have reported the combination of left predominant temporal atrophy and CTD [52, 53]. Recently, we described the clinical profile of rtvFTD and reported that slowness is a distinctive symptom of rtvFTD in particular in the later stages of the disease [9].Underlying tau pathology and MND form a potential explanation of this clinical observation.

One of our cases was found to harbour FTLD-FUS pathology. Consistent with the literature [15], FUS pathology is rare, and we show that the phenotype can also present as rtvFTD. In addition, another rtvFTD subject was diagnosed with FTLD-TDP type E in our cohort. TDP type E has been recently identified based on a small number of case series, and links to prominent behavioural and movement disturbances that was also consistent with our case [19].

This is the first study that systematically collected the underlying molecular neuropathology of rtvFTD, which challenges the assumption that rtvFTD is an FTLD-TDP type C disorder by reporting heterogeneous FTLD pathologies in the patients with rtvFTD. However, there are some limitations that need to be addressed. First of all, the number of our subjects was limited and the results mostly rely on the literature review. Secondly, current neuropathological criteria for FTLD could not be applied in all rtvFTD cases described in the literature.

The right temporal lobe plays a key role in memory, social cognition, verbal and especially non-verbal semantic cognition. Therefore, rtvFTD can present with a combination of psychiatric features and multi-domain cognitive impairment. Our results show that heterogeneous FTLD pathologies can initially cause right temporal lobe neurodegeneration and present with rtvFTD clinical features. To date, due to the lack of separate diagnostic criteria, rtvFTD has been relatively neglected in the large clinicopathological studies, although our findings of highly heterogeneous underlying pathologies in rtvFTD might have consequences for individualised patient management. Predominant semantic impairment associated with the predominant temporal lobe atrophy is related with the FTLD-TDP type C pathology whereas FTLD-tau is mostly related with behavioural problems and frontotemporal atrophy at the later stages of the disorder. Pyramidal and extrapyramidal disturbances are expected in rtvFTD not only in patients with FTLD-TDP type A/B, but also in FTLD-TDP type C and tau.

Our findings suggest that rtvFTD might be a separate pathological entity and future large scale studies are warranted to shed light on whether the presentation, disease course and associated pathology provide the evidence for this.

## Supplementary Information

**Additional file 1**. Details of the pathological examination.

**Additional file 2**. PRISMA flow diagram.

**Additional file 3**. Excluded studies.

## Data Availability

Data are available on request from the corresponding author.

## Abbreviations

AD: Alzheimer’s disease
AGD: Argyrophilic grain disease
ALS: Amyotrophic lateral sclerosis
bvFTD: Behavioural variant frontotemporal dementia
CBD: Corticobasal degeneration
FTD: Frontotemporal dementia
FTLD: Frontotemporal lobar degeneration
FUS: Fused in sarcoma
GGT: Globular glial tauopathy
MAPT: Microtubule associated protein tau
MND: Motor neuron degeneration
nfvPPA: Nonfluent variant primary progressive aphasia
PNFA: Progressive nonfluent aphasia
PSP: Progressive supranuclear palsy
PPA: Primary progressive aphasia
rtvFTD: Right temporal variant frontotemporal dementia
SD: Semantic dementia
svPPA: Semantic variant primary progressive aphasia
TDP-43: TAR DNA binding protein 43
