# Supplementary material for: Right temporal variant frontotemporal dementia is pathologically heterogeneous: a case-series and a systematic review
Source: Acta Neuropathol Commun. 2021 Aug 3;9:131. doi: 10.1186/s40478-021-01229-z (PMC8330072; doi:10.1186/s40478-021-01229-z)
Supplement: Supplementary file 1 — Additional file 1. Details of the pathological examination. [file 40478_2021_1229_MOESM1_ESM.docx]

**Supplementary material 1**

**Pathological examination**

Brain autopsy was carried out within 4 h of death according to the Legal and Ethical Code of Conduct of the Netherlands Brain Bank. Tissue blocks taken from all cortical areas, hippocampus, amygdala, basal ganglia, substantia nigra, pons, medulla oblongata, cerebellum and cervical spinal cord were embedded in paraffin blocks, and underwent routine staining with haematoxylin–eosin, Bodian, methenamine-silver and Congo red. Tissue blocks were taken from the right hemisphere in each case. Immunohistochemistry was performed using primary antibodies against hyperphosphorylated tau (AT8, Innogenetics; 1:40), ubiquitin (anti-ubiquitin, Dako; 1:500, following 80°C antigen retrieval), β-amyloid protein (anti-beta amyloid, Dako; 1:100, following formic acid pretreatment), α-synuclein (anti-α-synuclein, Zymed Laboratories; undiluted, following formic acid pretreatment), p62 (BD Biosciences Pharmingen; 1:200, following 80°C antigen retrieval), TDP-43 (Biotech; 1:100, following pressure cooking), TDP-43 phosphorylated at serine 409/410 (Cosmo Bio; 1:8000), fused in sarcoma (Sigma-Aldrich anti-fused in sarcoma; 1:25–200 with initial overnight incubation at room temperature, following pressure cooking). Primary antibodies were incubated overnight at 4°C. Endogenous peroxidase activity was inhibited by incubation in phosphate buffered saline–hydrogen peroxide–sodium azide solution (100 ml 0.1 M phosphate-buffered saline + 2 ml 30% H2O2 + 1 ml natriumazide) for 30 min. The Histostain-Plus broad-spectrum kit DAB (Zymed) was used, and slides were counterstained with Mayer's haematoxylin and mounted in Entellan®. The pathological diagnosis was made by an experienced neuropathologist (A.R.).
