# Supplementary material for: Right temporal variant frontotemporal dementia is pathologically heterogeneous: a case-series and a systematic review
Source: Acta Neuropathol Commun. 2021 Aug 3;9:131. doi: 10.1186/s40478-021-01229-z (PMC8330072; doi:10.1186/s40478-021-01229-z)
Supplement: Supplementary file 2 — Additional file 2. PRISMA flow diagram. [file 40478_2021_1229_MOESM2_ESM.doc]

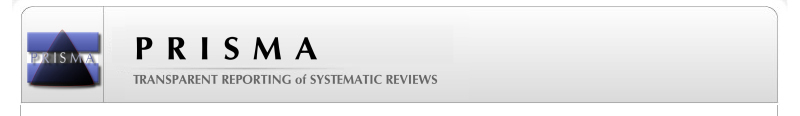
**PRISMA 2009 Flow Diagram**

**Screening**

**Included**

**Eligibility**

**Identification**

Records identified through database searching
(n =9743 )

Additional records identified through other sources
(n = 0 )

Records after duplicates removed
(n =2338 )

Records screened
(n = 2338 )

Records excluded
(n = 1935 )

Full-text articles assessed for eligibility
(n = 403 )

Full-text articles excluded, with reasons
(n =372 )

Studies included in qualitative synthesis
(n = 34 )

Studies included in quantitative synthesis (meta-analysis)
(n = 21 )
