# Supplementary material for: Right temporal variant frontotemporal dementia is pathologically heterogeneous: a case-series and a systematic review
Source: Acta Neuropathol Commun. 2021 Aug 3;9:131. doi: 10.1186/s40478-021-01229-z (PMC8330072; doi:10.1186/s40478-021-01229-z)
Supplement: Supplementary file 3 — Additional file 3. Excluded studies. [file 40478_2021_1229_MOESM3_ESM.docx]

**Supplementary material 3**

All reviewed articles were re-reviewed. A number of the studies were excluded based on authors consensus.

|  | Publications | N | Institution | Country | Status |
| --- | --- | --- | --- | --- | --- |
| 1 | (Miki *et al.*, 2019) | 1 | UCL | UK | Included |
| 2 | (Snowden *et al.*, 2019) | 1 | MCCN | UK | Included |
| 3 | (Kim *et al.*, 2018b) | 1 | Northwestern  University | USA | Excluded: A logopenic variant PPA subject with right hemisphere language dominance |
| 4 | (Caplan *et al.*, 2018) | 9 | UCSF | USA | Included |
| 5 | (Irwin *et al.*, 2018) | 3 | Penn FTD Center | USA | Excluded: Insufficient radiological data to identify the right predominant temporal lobar atrophy |
| 6 | (Kim *et al.*, 2018a) | 1 | UCSF | USA | Included |
| 7 | (Perry *et al.*, 2017) | 9 | UCSF | USA | Excluded: Case duplication. Study design; centre/period: UCSF Memory and Aging Center/ from 1998 to 2012. Caplan *et al.,* (2018) covers these patients |
| 8 | (Koriath *et al.*, 2017) | 1 | UCL | UK | Included |
| 9 | (Kuuluvainen *et al.*, 2017) | 1 | Helsinki University | Finland | Included |
| 10 | (Wood *et al.*, 2016) | 1 | Cambridge Brain Bank | UK | Included |
| 11 | (Clark *et al.*, 2015) | 1 | UCL | UK | Included |
| 12 | (Moreno *et al.*, 2015) | 1 | UCSF | USA | Included |
| 13 | (Henry *et al.*, 2014) | 1 | UCSF | USA | Excluded: Case duplication. Study design; centre/ period: UCSF Memory and Aging Center/ 2006. Caplan *et al.,* (2018) covers this patient |
| 14 | (Cannon *et al.*, 2013) | 1 | Mayo Clinic | USA | Excluded: A case with right temporal atrophy, however, clinical features are suggestive for ALS rather than FTD |
| 15 | (Josephs *et al.*, 2013) | 7 | Mayo Clinic | USA | Included |
| 16 | (Coon *et al.*, 2012) | 2 | Mayo Clinic | USA | Included |
| 17 | (Lee *et al.*, 2012) | 1 | UCSF | USA | Included |
| 18 | (Laforce *et al.*, 2012) | 1 | UCSF | USA | Excluded: Frontal atrophy scores are equal or higher than right temporal atrophy scores |
| 19 | (Mahoney *et al.*, 2012) | 1 | UCL | UK | Excluded: Individual pathological data are not available |
| 20 | (Rohrer *et al.*, 2011) |  | UCL | UK | Excluded: Individual clinical and radiological data are not available |
| 21 | (Ostberg and Bogdanovic, 2011) | 1 | Uppsala University | Sweden | Included |
| 22 | (Mimuro *et al.*, 2010) | 1 | Aichi University | Japan | Excluded: Patient is cognitively normal |
| 23 | (Kelley *et al.*, 2010) | 1 | UCL | UK | Included |
| 24 | (Kobayashi *et al.*, 2010) | 1 | Tokyo IP | Japan | Included |
| 25 | (Kuwahara *et al.*, 2010) | 1 | Tokyo IP | Japan | Included |
| 26 | (Rohrer *et al.*, 2010) | 4 | UCSF | USA | Excluded: Case duplication. Study design; centre/ period: UCSF Memory and Aging Center/ unknown. Caplan *et al.,* (2018) covers these patients |
| 27 | (Chan *et al.*, 2009) | 2 | UCL | UK | 1 case included, 1 case excluded (lack of TDP-43 staining) |
| 28 | (Josephs *et al.*, 2009) | 11 | Mayo clinic | USA | 8 cases included, 3 cases excluded (case duplication). Study design; centre/ period: Mayo Clinic Alzheimer Disease Research Center or Alzheimer Disease Patient Registry/ from January 1992 to December 2008. Josephs *et al.,* (2013) covers these 3 cases with TDP type C pathology |
| 29 | (Kelley *et al.*, 2009) | 2 | UCL | UK | Included |
| 30 | (Yoshida, 2009) | 1 | Aichi University | Japan | Included |
| 31 | (Beck *et al.*, 2008) | 3 | UCL | UK | Excluded: Frontal atrophy scores are equal or higher than right temporal atrophy scores |
| 32 | (Leverenz *et al.*, 2007) | 1 | VAPSHCS | USA | Excluded: Frontal atrophy scores are equal or higher than right temporal atrophy scores |
| 33 | (Davion *et al.*, 2007) | 1 | Northwestern  University | USA | Included |
| 34 | (Davies *et al.*, 2005) | 2 | University of Cambridge and University of Sydney | UK, Australia | Excluded due to lack of TDP-43 staining |

UCL: University College London; UCSF: University of California San Francisco; FTD: frontotemporal dementia; MCCN: Manchester Centre for Clinical Neurosciences; IP: institute of psychiatry; VAPSHCS: Veterans Affairs Puget Sound Health Care System; ALS: amyotrophic lateral sclerosis; TDP-43: TAR DNA binding protein 43.

Beck J, Rohrer JD, Campbell T, Isaacs A, Morrison KE, Goodall EF*, et al.* A distinct clinical, neuropsychological and radiological phenotype is associated with progranulin gene mutations in a large UK series. Brain 2008; 131(Pt 3): 706-20.

Cannon A, Fujioka S, Rutherford NJ, Ferman TJ, Broderick DF, Boylan KB*, et al.* Clinicopathologic variability of the GRN A9D mutation, including amyotrophic lateral sclerosis. Neurology 2013; 80(19): 1771-7.

Caplan A, Marx G, Elofson J, Lis C, Grinberg L, Miller B*, et al.* A case of semantic variant primary progressive aphasia with Pick's pathology. Neurocase 2018; 24(2): 90-4.

Chan D, Anderson V, Pijnenburg Y, Whitwell J, Barnes J, Scahill R*, et al.* The clinical profile of right temporal lobe atrophy. Brain 2009; 132(Pt 5): 1287-98.

Clark CN, Lashley T, Mahoney CJ, Warren JD, Revesz T, Rohrer JD. Temporal Variant Frontotemporal Dementia is Associated with Globular Glial Tauopathy. Cogn Behav Neurol 2015; 28(2): 92-7.

Coon EA, Whitwell JL, Parisi JE, Dickson DW, Josephs KA. Right temporal variant frontotemporal dementia with motor neuron disease. J Clin Neurosci 2012; 19(1): 85-91.

Davies RR, Hodges JR, Kril JJ, Patterson K, Halliday GM, Xuereb JH. The pathological basis of semantic dementia. Brain 2005; 128(Pt 9): 1984-95.

Davion S, Johnson N, Weintraub S, Mesulam MM, Engberg A, Mishra M*, et al.* Clinicopathologic correlation in PGRN mutations. Neurology 2007; 69(11): 1113-21.

Henry ML, Wilson SM, Ogar JM, Sidhu MS, Rankin KP, Cattaruzza T*, et al.* Neuropsychological, behavioral, and anatomical evolution in right temporal variant frontotemporal dementia: a longitudinal and post-mortem single case analysis. Neurocase 2014; 20(1): 100-9.

Irwin DJ, McMillan CT, Xie SX, Rascovsky K, Van Deerlin VM, Coslett HB*, et al.* Asymmetry of post-mortem neuropathology in behavioural-variant frontotemporal dementia. Brain 2018; 141(1): 288-301.

Josephs KA, Whitwell JL, Knopman DS, Boeve BF, Vemuri P, Senjem ML*, et al.* Two distinct subtypes of right temporal variant frontotemporal dementia. Neurology 2009; 73(18): 1443-50.

Josephs KA, Whitwell JL, Murray ME, Parisi JE, Graff-Radford NR, Knopman DS*, et al.* Corticospinal tract degeneration associated with TDP-43 type C pathology and semantic dementia. Brain 2013; 136(Pt 2): 455-70.

Kelley BJ, Haidar W, Boeve BF, Baker M, Graff-Radford NR, Krefft T*, et al.* Prominent phenotypic variability associated with mutations in Progranulin. Neurobiol Aging 2009; 30(5): 739-51.

Kelley BJ, Haidar W, Boeve BF, Baker M, Shiung M, Knopman DS*, et al.* Alzheimer disease-like phenotype associated with the c.154delA mutation in progranulin. Arch Neurol 2010; 67(2): 171-7.

Kim EJ, Brown JA, Deng J, Hwang JL, Spina S, Miller ZA*, et al.* Mixed TDP-43 proteinopathy and tauopathy in frontotemporal lobar degeneration: nine case series. J Neurol 2018a; 265(12): 2960-71.

Kim G, Vahedi S, Gefen T, Weintraub S, Bigio EH, Mesulam MM*, et al.* Asymmetric TDP pathology in primary progressive aphasia with right hemisphere language dominance. Neurology 2018b; 90(5): e396-e403.

Kobayashi Z, Tsuchiya K, Arai T, Yokota O, Yoshida M, Shimomura Y*, et al.* Clinicopathological characteristics of FTLD-TDP showing corticospinal tract degeneration but lacking lower motor neuron loss. J Neurol Sci 2010; 298(1-2): 70-7.

Koriath CA, Bocchetta M, Brotherhood E, Woollacott IO, Norsworthy P, Simon-Sanchez J*, et al.* The clinical, neuroanatomical, and neuropathologic phenotype of TBK1-associated frontotemporal dementia: A longitudinal case report. Alzheimers Dement (Amst) 2017; 6: 75-81.

Kuuluvainen L, Poyhonen M, Pasanen P, Siitonen M, Rummukainen J, Tienari PJ*, et al.* A Novel Loss-of-Function GRN Mutation p.(Tyr229*): Clinical and Neuropathological Features. J Alzheimers Dis 2017; 55(3): 1167-74.

Kuwahara H, Tsuchiya K, Saito Y, Kobayashi Z, Miyazaki H, Izumiyama Y*, et al.* Frontotemporal lobar degeneration with motor neuron disease showing severe and circumscribed atrophy of anterior temporal lobes. J Neurol Sci 2010; 297(1-2): 92-6.

Laforce R, Jr., Kerchner GA, Rabinovici GD, Fong JC, Miller BL, Seeley WW*, et al.* A 44-year-old man with profound behavioural changes. Can J Neurol Sci 2012; 39(4): 527-30.

Lee SE, Seeley WW, Poorzand P, Rademakers R, Karydas A, Stanley CM*, et al.* Clinical characterization of bvFTD due to FUS neuropathology. Neurocase 2012; 18(4): 305-17.

Leverenz JB, Yu CE, Montine TJ, Steinbart E, Bekris LM, Zabetian C*, et al.* A novel progranulin mutation associated with variable clinical presentation and tau, TDP43 and alpha-synuclein pathology. Brain 2007; 130(Pt 5): 1360-74.

Mahoney CJ, Beck J, Rohrer JD, Lashley T, Mok K, Shakespeare T*, et al.* Frontotemporal dementia with the C9ORF72 hexanucleotide repeat expansion: clinical, neuroanatomical and neuropathological features. Brain 2012; 135(Pt 3): 736-50.

Miki Y, Ling H, Crampsie S, Mummery CJ, Rohrer JD, Jaunmuktane Z*, et al.* Corticospinal tract degeneration and temporal lobe atrophy in frontotemporal lobar degeneration TDP-43 type C pathology. Neuropathol Appl Neurobiol 2019.

Mimuro M, Yoshida M, Miyao S, Harada T, Ishiguro K, Hashizume Y. Neuronal and glial tau pathology in early frontotemporal lobar degeneration-tau, Pick's disease subtype. J Neurol Sci 2010; 290(1-2): 177-82.

Moreno F, Rabinovici GD, Karydas A, Miller Z, Hsu SC, Legati A*, et al.* A novel mutation P112H in the TARDBP gene associated with frontotemporal lobar degeneration without motor neuron disease and abundant neuritic amyloid plaques. Acta Neuropathol Commun 2015; 3: 19.

Ostberg P, Bogdanovic N. Semantic dementia with lower motor neuron disease showing FTLD-TDP type 3 pathology (sensu Mackenzie). Neuropathology 2011; 31(3): 271-9.

Perry DC, Brown JA, Possin KL, Datta S, Trujillo A, Radke A*, et al.* Clinicopathological correlations in behavioural variant frontotemporal dementia. Brain 2017; 140(12): 3329-45.

Rohrer JD, Geser F, Zhou J, Gennatas ED, Sidhu M, Trojanowski JQ*, et al.* TDP-43 subtypes are associated with distinct atrophy patterns in frontotemporal dementia. Neurology 2010; 75(24): 2204-11.

Rohrer JD, Lashley T, Schott JM, Warren JE, Mead S, Isaacs AM*, et al.* Clinical and neuroanatomical signatures of tissue pathology in frontotemporal lobar degeneration. Brain 2011; 134(Pt 9): 2565-81.

Snowden JS, Kobylecki C, Jones M, Thompson JC, Richardson AM, Mann DMA. Association between semantic dementia and progressive supranuclear palsy. J Neurol Neurosurg Psychiatry 2019; 90(1): 115-7.

Wood R, Moodley K, Hodges JR, Allinson K, Spillantini MG, Chan D. Slowly progressive behavioural presentation in two UK cases with the R406W MAPT mutation. Neuropathol Appl Neurobiol 2016; 42(3): 291-5.

Yoshida M, Kono, K., Nomura, M., Hashizume, Y. An autopsy case with semantic dementia, coexistence TDP-43-positive FTLD-U and Alzheimer disease pathology. Japanese Society of Neuropathology - Abstracts of the 50th Annual Meeting. Takamatsu, Japan: Elsevier; 2009. p. 370.
